# Estimation of Vehicle Attitude, Acceleration, and Angular Velocity Using Convolutional Neural Network and Dual Extended Kalman Filter

**DOI:** 10.3390/s21041282

**Published:** 2021-02-11

**Authors:** Minseok Ok, Sungsuk Ok, Jahng Hyon Park

**Affiliations:** 1 Department of Automotive Electronics & Control Engineering, Hanyang University, Seoul 04763, Korea; doree81@hanyang.ac.kr; 2 Autonomous Driving Center, Hyundai Motor Company R&D Division, Seoul 06182, Korea; sungsuk.ok@hyundai.com

**Keywords:** sensor fusion, state estimation, vehicle dynamics, convolutional neural network, dual extended Kalman filter, vehicle roll and pitch angle, vehicle acceleration and angular velocity

## Abstract

The acceleration of a vehicle is important information in vehicle states. The vehicle acceleration is measured by an inertial measurement unit (IMU). However, gravity affects the IMU when there is a transition in vehicle attitude; thus, the IMU produces an incorrect signal output. Therefore, vehicle attitude information is essential for obtaining correct acceleration information. This paper proposes a convolutional neural network (CNN) for attitude estimation. Using sequential data of a vehicle’s chassis sensor signal, the roll and pitch angles of a vehicle can be estimated without using a high-cost sensor such as a global positioning system or a six-dimensional IMU. This paper also proposes a dual-extended Kalman filter (DEKF), which can accurately estimate acceleration/angular velocity based on the estimated roll/pitch information. The proposed method is validated by real-car experiment data and CarSim, a vehicle simulator. It accurately estimates the attitude estimation with limited sensors, and the exact acceleration/angular velocity is estimated considering the roll and pitch angle with de-noising effect. In addition, the DEKF can improve the modeling accuracy and can estimate the roll and pitch rates.

## 1. Introduction

In recent decades, the vehicle controller has been developed significantly for stability and user convenience. Typically, the electronic stability controller (ESC) or active roll stabilization (ARS) are used for ensuring vehicle stability in chassis controller, adaptive cruise control (ACC), or lane keeping system (LKS) as well as for ensuring convenience in advanced driver assistant system (ADAS). To improve the performance of these controllers, vehicle states must be estimated with a high accuracy. The commonly required and important state information of aforementioned controllers are acceleration and angular velocity. In a vehicle, the inertial measure unit (IMU) measures the acceleration and angular velocity using an inertial force. However, if a transition in attitude (roll and pitch) occurs, the gravitational force is reflected in the sensor value. The IMU cannot distinguish between gravitational force and inertial forces; therefore, a change in the attitude of the vehicle causes a fatal error in the IMU.

Several studies have been conducted to overcome the errors in these accelerometers and to estimate the exact state of the vehicle. In [1,2], the adaptive Kalman filter was designed to minimize the effect of the accelerometer offset errors. In addition, the Kalman filter was used to estimate the accelerometer offset and vehicle velocity [3,4,5]. However, the accuracy of estimating vehicle states was limited because of a lack of information of the vehicle attitude.

Several studies have been conducted to estimate the accurate vehicle attitude in various ways. Using IMU or GPS or vehicle dynamics, researchers tried to estimate the vehicle roll and pitch angle. In addition, due to the recent developments in artificial intelligence and neural networks, data-driven estimators have been used for state estimation [6]. These estimators have the advantage of having high accuracy by training the data directly in situations where accurate mathematical modeling is difficult. The literature review is discussed separately in Section 2.

**Figure 1:**
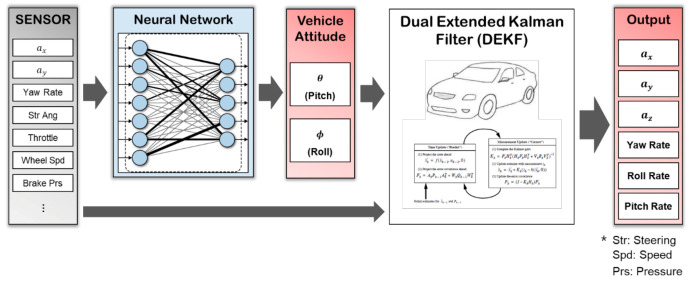
Architecture overview.

This paper proposes a novel vehicle attitude estimator using a convolutional neural network (CNN), based on the advantages of a data-driven estimator. Figure 1 shows the architecture of the proposed algorithm. First, we select the features based on vehicle roll and pitch dynamics, importance judgment and sensor usability. By using the selected features sequentially [7], the three-dimensional IMU and vehicle sensor build a CNN-based regression model that can estimate the vehicle roll and pitch angles without GPS. Moreover, a dual extended Kalman filter (DEKF) is designed to estimate the exact acceleration/angular velocity based on the estimated roll and pitch angles with removing the effect of gravitational force. The proposed model is based on the sprung mass six-degree-of-freedom (6-DOF) model and increases the modeling accuracy while estimating the tire cornering stiffness simultaneously. The performance of the proposed algorithm was verified using real car experiment data and MATLAB/Simulink with CarSim, a vehicle simulator. A CNN conducted training and verification using data collected from various driving conditions as an experimental vehicle with RT-3002 which is a high-accuracy GPS-inertial navigation system (INS) from Oxford Technical Solutions Ltd. The performance of the DEKF was verified with a scenario in which postural changes occur using CarSim and MATLAB/Simulink.

The remainder of this paper is organized as follows. Section 2 describes the related literature review. Section 3 explains the methodology including neural network and DEKF. Section 4 describes the results of performance verification. Section 5 summarizes the study and contributions.

## 2. Literature Review

Several studies have been conducted to estimate the accurate vehicle attitude in various ways. In [8], authors proposed an observer which can estimate the land vehicle’s roll and pitch by using an IMU and the kinematics model. Also, the adaptive Kalman filter was proposed based on IMU aided by vehicle dynamics [9]. However, these methods have a low accuracy in dynamic situations. This problem was attempted to be solved in [10,11,12,13], based on the Kalman filter by compensating for the external acceleration that interfered with the estimation of the attitude, but the accuracy was limit.

Therefore, many researchers have proposed sensor fusion method using not only IMU but fusion with global positioning system (GPS). Representatively, the method in which IMU and GPS fusion with Kalman filter [14,15,16] or sliding mode observer [17] can be used. These methods can increase the accuracy, but they require high-cost sensors such as GPS and are highly affected by GPS performance.

Accordingly, vehicle attitude estimator studies were conducted without the use of GPS, based on the characteristics of the vehicle dynamics. Using vehicle roll dynamics, a dynamic observer design based on a reliable rollover index [18] was proposed. Also, the Kalman filter [19] and the robust observer [20] based on vehicle roll dynamics which estimate roll angle were proposed. Alternatively, using lateral dynamics, the research which estimate road bank angle [21,22] was proposed. However, these methods exhibited limited accuracy in transient situations. A method for estimating both roll and pitch attitudes using a six-dimensional IMU and bicycle model [23,24] was also studied. These methods exhibited a high accuracy, but they have a short validation range and required hard-to-get data such as six-dimensional IMU.

Several recent studies have also been conducted to estimate the vehicle roll and pitch angle using neural network. In [25,26,27], the vehicle roll angle estimation method using sensor fusion with a neural network and Kalman filter was proposed. Furthermore, a vehicle roll and road bank angle estimation method based on a deep neural network was introduced [28]. However, the pitch attitudes were not estimated, and the six-dimensional IMU data such as roll rate or vertical acceleration were required, which complicated the estimation. Literature reviews are summarized in Table 1.

## 3. Methodology

### 3.1. Data-Driven-Based Estimator (Neural Network) Design

#### 3.1.1. Feature Selection

Feature selection is based on vehicle dynamics, sensor usability, and attention mechanism. The purpose of neural network is to design a regression model for calculating the roll and pitch angles, so select the primary feature candidates for the vehicle’s roll and pitch dynamics. Vehicle roll dynamics [29] can be written as:

(1) $$\Sigma M_{x}=h_{R}m_{s}gsin\phi -\frac{1}{2}k_{s}l_{s}^{2}sin\phi -\frac{1}{2}b_{s}l_{s}^{2}\dot{\phi }sin\phi +\Sigma F_{y}\left(h_{CG}-h_{R}\right)$$

(2) $$\Sigma F_{y}=2C_{\alpha f}\left(\delta -\frac{v_{y}+l_{f}\dot{\psi }}{v_{x}}\right)+2C_{\alpha r}\left(-\frac{v_{y}-l_{r}\dot{\psi }}{v_{x}}\right)+m_{s}gsin\phi .\;$$

where $M_{x}$ is the moment respect to the roll axis, $h_{R}$ is the height of the from center of gravity to roll center, $m_{s}$ is the vehicle sprung mass, g is the gravity acceleration, ϕ is the roll of vehicle, $k_{s}$ is the stiffness of the suspension, $l_{s}$ is the length of the wheel track, $b_{s}$ is the damping coefficient of the suspension, and $h_{CG}$ is the height of the center of gravity from the ground.

The lateral force applied to the vehicle can be expressed by (2) based on the bicycle model. $C_{\alpha f}$ and $C_{\alpha r}$ are the cornering stiffness values of each front and rear tire, respectively, δ is the steering angle, $v_{x}$ and $v_{y}$ are the velocities of each *x* and *y* axes, respectively, $l_{f}$ and $l_{r}$ are the length of each front and rear wheel axis from the center of gravity, and $\dot{\psi }$ is the yaw rate.

The vehicle pitch dynamics can be written as [29]:

(3) $$\Sigma M_{y}=-k_{s}l_{f}^{2}sin\theta -k_{s}l_{r}^{2}sin\theta -b_{s}l_{f}^{2}\dot{\theta }cos\theta -b_{s}l_{r}^{2}\dot{\theta }cos\theta -\left(\Sigma F_{drive}-\Sigma F_{brake}\right)h_{CG}$$

(4) $$\Sigma F_{drive}=\left[\frac{N_{tf}\eta_{tf}}{r_{eff}}T_{e}-\left\{\left(I_{e}+I_{t}\right)N_{tf}^{2}+I_{d}N_{f}^{2}+I_{w}\right\}\frac{a_{x}}{r_{eff}^{2}}\right]$$

(5) $$\Sigma F_{brake}=\frac{2P_{brake}A_{caliper}\mu_{pad}R_{b}}{r_{eff}}$$

where $M_{y}$ is the moment with respect to the pitch axis and θ is the pitch of the vehicle.

The traction force applied to the vehicle can be expressed by (4) based on the vehicle drive-line dynamics. $N_{tf}$ is the gear ratio of the transmission and differential gears, $\eta_{tf}$ is the efficiency of the power transfer from the engine to the wheel axis, $r_{eff}$ is the effective radius of the tire, $T_{e}$ is the engine torque, $I_{e},\;I_{t},\;I_{d},\;I_{w}$ are the inertia of each engine, transmission, differential gear, and wheel, respectively, and $a_{x}$ is the longitudinal acceleration of the vehicle.

Brake force applied to the vehicle is given by (5). $P_{brake}$ is the pressure of the master cylinder, $A_{caliper}$ is the area of the brake pad caliper, $\mu_{pad}$ is the friction coefficient of the brake pad, and $R_{b}$ is the distance from the wheel center to the brake pad.

Based on (1)–(5), we can choose only variables, except for static parameters such as the vehicle mass. As a result, a total of 13 feature candidates were selected, including the acceleration and yaw rate. Subsequently, seven features were selected considering the availability of sensors in the experimental vehicle. Table 2 shows the selected features. Based on these, we conducted an analysis of importance based on the attention mechanism [30]. The results of the importance analysis are presented in Appendix A. According to Appendix A, the final features were selected same as Table 2.

#### 3.1.2. Network Design

Before configuring a 2-d input to the CNN, we calculate $\phi_{static,pseudo}$ and $\theta_{static,pseudo}$ reflecting the static roll and pitch angles using feature sensor values. The pseudo values are written as:

(6) $$\phi_{static,pseudo}=e^{-a\cdot V_{x}}\cdot \sin^{-1}\left(\frac{a_{y}}{g}\right)$$

(7) $$\theta_{static,pseudo}=e^{-b\cdot V_{x}}e^{-c\cdot \theta_{throttle}}e^{-\frac{d}{P_{brake}}}\cdot \sin^{-1}\left(-\frac{a_{x}}{g}\right)$$

where a,b,c,d are the constant design parameters. (5) and (6) are similar to the road bank and the slope angle, respectively when the vehicle’s wheel speed is zero. If the vehicle’s wheel speed increases, (5) and (6) become zero. 

**Figure 2:**
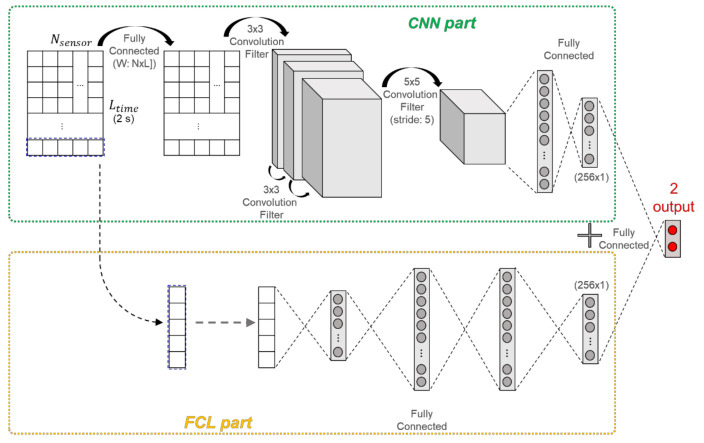
Neural network architecture.

The neural network architecture is shown in Figure 2. The network is composed of CNN part and fully connected layer (FCL) part in parallel configuration. The input array is composed of a mux of each sequential feature sensor data including (5), (6). The sequential information is 2 s for 0.01 s, therefore the input array size is (200 × 9). CNN part uses all inputs, namely all-time series data in the past 2 s, but FCL part uses only the last row of the input array, meaning only current step data. This means that the CNN part is designed with the intention of estimating dynamic vehicle body changes while driving and FCL part is designed to estimate the roll and pitch angles in static scenarios. The CNN part comprises four convolution layers and two fully connected layers. The first layer converts the input matrix into a square matrix and shuffles the sensor placement order. Then, it passes through the three convolution layers, and then pass the one convolution layer with large size filter and wide strides for compressing the data. Next, unfold to fully connected layer and make the last layer’s size (256 × 1). In FCL part, there are four layers and final layer’s size is also (256 × 1). The two final layers are concatenated, and then the regression model is constructed, which calculates two outputs using one fully connected layer. Hyperparameters of the neural network such as the number of filters or activation function are described in Appendix B.

### 3.2. Dual Extended Falman Filter Design

#### 3.2.1. State Space Model

**Figure 3:**
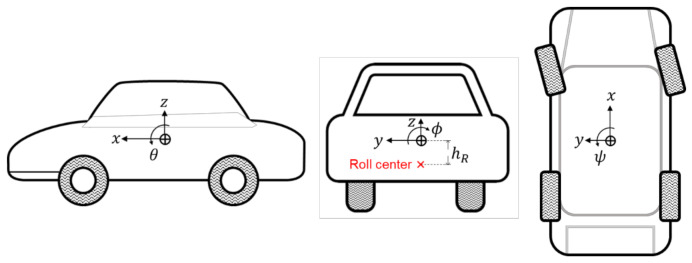
Vehicle sprung mass 6 degree of freedom motion.

The state space model is based on the vehicle 6-DOF sprung mass model [31,32] for expressing the six-dimensional acceleration and angular velocity. The dynamics are composed based on the Euler rigid body equation and the force or moment of each axis which is given by each dynamic [29]. Figure 3 shows the six-dimensional motion of vehicle sprung mass. The forces or moment of each axis are given by:

(8) $$\Sigma F_{x}=m_{s}\left(\dot{v_{x}}+v_{z}\dot{\theta }-v_{y}\dot{\psi }-h_{R}\ddot{\theta }+h_{R}\dot{\phi }\dot{\psi }\right)$$

(9) $$\Sigma F_{y}=m_{s}\left(\dot{v_{y}}+v_{x}\dot{\psi }-v_{z}\dot{\phi }-h_{R}\ddot{\phi }+h_{R}\dot{\theta }\dot{\psi }\right)$$

(10) $$\Sigma F_{z}=m_{s}\left(\dot{v_{z}}+v_{y}\dot{\phi }-v_{x}\dot{\theta }-h_{R}{\dot{\phi }}^{2}-h_{R}{\dot{\theta }}^{2}\right)$$

(11) $$\Sigma M_{x}=I_{x}\ddot{\phi }+\left(I_{z}-I_{y}\right)\dot{\theta }\dot{\psi }-m_{s}h_{R}\left(\dot{v_{y}}+v_{x}\dot{\psi }-v_{z}\dot{\phi }-h_{R}\ddot{\phi }+h_{R}\dot{\theta }\dot{\psi }\right)$$

(12) $$\Sigma M_{y}=I_{y}\ddot{\theta }+\left(I_{x}-I_{z}\right)\dot{\phi }\dot{\psi }$$

(13) $$\Sigma M_{z}=I_{z}\ddot{\psi }+\left(I_{y}-I_{x}\right)\dot{\phi }\dot{\theta }$$

where $\Sigma F_{x},\;\Sigma F_{y},\;\Sigma F_{z}$ are the sum of forces of the *x*, *y*, and *z* axes, respectively. $\Sigma M_{x},\;\Sigma M_{y},\;\Sigma M_{z}$ are the sum of moments of the *x*, *y*, and *z* axes, respectively. $I_{x},\;I_{y},\;I_{z}$ are the moments of inertia along the *x*, *y*, and *z* axes, respectively. $\Sigma F_{x},\;\Sigma F_{y},\;\Sigma F_{z},\;\Sigma M_{x},\;\Sigma M_{y},$ and $\Sigma M_{z}$ can be derived from a vehicle model such as a bicycle model. Therefore, the nonlinear state space equation can be written as:

(14) $${\dot{x}}_{1}=\frac{\left(\frac{N_{tf}\eta_{tf}}{r_{eff}}u_{1}-\Sigma F_{brake}-\frac{1}{2}\rho C_{d}Ax_{1}^{2}-m_{s}gsin\left(x_{8}\right)\right)}{\left\{m_{s}+\frac{\left(I_{e}+I_{t}\right)N_{tf}^{2}+I_{d}N_{f}^{2}+I_{w}}{r_{eff}^{2}}\right\}}-x_{3}x_{5}+x_{2}x_{6}+h_{R}\dot{x_{5}}-h_{R}x_{4}x_{6}$$

(15) $${\dot{x}}_{2}=\frac{1}{m_{s}}\left\{\Sigma F_{y}+m_{s}gsin\left(x_{7}\right)\right\}-x_{1}x_{6}+x_{3}x_{4}+h_{R}\dot{x_{4}}-h_{R}x_{5}x_{6}$$

(16) $${\dot{x}}_{3}=\frac{1}{m_{s}}k_{s}\sin\left(x_{8}\right)\left(l_{f}-l_{r}\right)+b_{s}\cos\left(x_{8}\right)x_{5}\left(l_{f}-l_{r}\right)-x_{2}x_{4}+x_{1}x_{5}+h_{R}x_{4}^{2}+h_{R}x_{5}^{2}$$

(17) $${\dot{x}}_{4}=\frac{1}{I_{x}+m_{s}h_{R}^{2}}\left\{h_{R}m_{s}gsin\left(x_{7}\right)-\frac{1}{2}k_{s}l_{s}^{2}\sin\left(x_{7}\right)-\frac{1}{2}b_{s}l_{s}^{2}x_{4}\cos\left(x_{7}\right)+\Sigma F_{y}\left(h_{CG}-h_{R}\right)-\left(I_{z}-I_{y}\right)x_{5}x_{6}+m_{s}h_{R}\left({\dot{x}}_{2}+x_{1}x_{6}-x_{3}x_{4}+h_{R}x_{5}x_{6}\right)\right\}$$

(18) $${\dot{x}}_{5}=\frac{1}{I_{y}}\left\{-\left(l_{f}^{2}+l_{r}^{2}\right)k_{s}\sin\left(x_{8}\right)-\left(l_{f}^{2}+l_{r}^{2}\right)b_{s}x_{5}\cos\left(x_{8}\right)-\left(\Sigma F_{drive}-\Sigma F_{brake}\right)h_{CG}-\left(I_{x}-I_{z}\right)x_{5}x_{6}\right\}$$

(19) $${\dot{x}}_{6}=\frac{1}{I_{z}}\left\{-\frac{2l_{f}C_{\alpha f}-2l_{r}C_{\alpha r}}{x_{1}}x_{2}-\frac{2l_{f}^{2}C_{\alpha f}+2l_{r}^{2}C_{\alpha r}}{x_{1}}x_{6}+2l_{f}C_{\alpha f}u_{3}-\left(I_{y}-I_{x}\right)x_{4}x_{5}\right\}$$

(20) $${\dot{x}}_{7}=x_{4}$$

(21) $${\dot{x}}_{8}=x_{5}$$

$\Sigma F_{drive}$, $\Sigma F_{brake}$, and $\Sigma F_{y}$ have been described previously. The state vector $x=\left[v_{x}\;v_{y}\;v_{z}\;\dot{\phi }\;\dot{\theta }\;\dot{\psi }\;\phi \;\theta \right]$, state input $u=\;\left[T_{e}\;P_{brake}\;\delta \right]$. ρ is the density of the air, $C_{d}$ is the drag coefficient and A is the frontal area of the vehicle.

State outputs include longitudinal acceleration, lateral acceleration, and yaw rate from IMU. The roll and pitch angles from the neural network are also included. Thus, the state output $y=\left[a_{x}\;a_{y}\;\dot{\psi }\;\phi \;\theta \right]$ and it can be written as:

(22) $$a_{x}=\frac{\left(\frac{N_{tf}\eta_{tf}}{r_{eff}}u_{1}-\Sigma F_{brake}-\frac{1}{2}\rho C_{d}Ax_{1}^{2}-m_{s}gsin\left(x_{8}\right)\right)}{\left\{m_{s}+\frac{\left(I_{e}+I_{t}\right)N_{tf}^{2}+I_{d}N_{f}^{2}+I_{w}}{r_{eff}^{2}}\right\}}$$

(23) $$a_{y}=\frac{1}{m_{s}}\left\{\Sigma F_{y}+m_{s}gsin\left(x_{7}\right)\right\}$$

(24) $$\dot{\psi }=x_{6}$$

(25) $$\phi =x_{7}$$

(26) $$\theta =x_{8}$$

#### 3.2.2. Observability Check

Before designing the estimator, the observability must be checked. The observability of the nonlinear state space model can be checked by the Lie derivative [33]. When the state space equation is expressed as $\dot{x}=f\left(x,u\right),\;y=h\left(x\right)$, the Lie derivative and observability matrix can be written as:

(27) $$L_{f}^{0}=h\left(x\right)\;L_{f}^{k+1}=\frac{\partial L_{f}^{k}}{\partial x}f=\nabla L_{f}^{k}\cdot f$$

(28) $$O={\left[\begin{array}{c} \nabla L_{f}^{0}\\ \nabla L_{f}^{1}\\ \vdots \\ \nabla L_{f}^{n-1} \end{array}\right]}_{x=x_{0}}$$

where O is the observability matrix. Using the rank of the observability matrix, the system’s observability can be seen locally. As a result of checking the rank of the observability matrix, the observability matrix has full rank in range of $v_{x}\neq 0$; therefore, this system is locally observable for the range except $v_{x}=0$.

#### 3.2.3. Dual Extended Kalman Filter Module

Among the vehicle dynamics parameters, the cornering stiffness varies under conditions such as the vehicle load. To reduce the errors while modeling, the cornering stiffness should be estimated.

**Figure 4:**
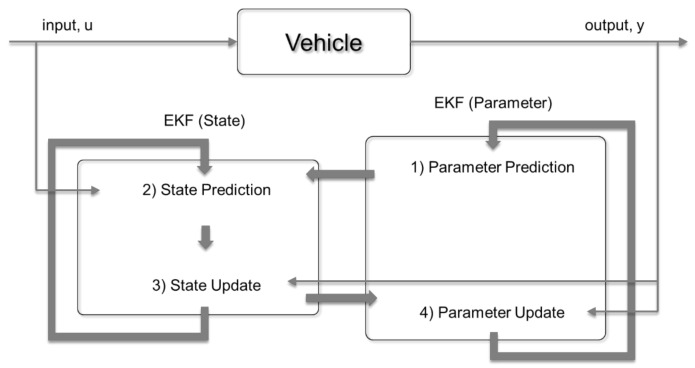
Scheme of dual-extended Kalman filter (DEKF).

This study adopts the DEKF as an estimator for reducing the error and increasing the modeling accuracy. Figure 4 shows the DEKF scheme. The state vector, state input, state output, and state space equation have been discussed in Section 3.2.1. The DEKF module works according to the following recursive algorithm [34]:

Parameter prediction:

(29) $${\hat{x}}_{p}^{-}\left(t\right)\;={\hat{x}}_{p}\left(t-1\right)$$

(30) $$P_{p}^{-}\left(t\right)\;=P_{p}\left(t-1\right)+Q_{p}$$

State prediction:

(31) $${\hat{x}}_{s}^{-}\left(t\right)\;=f\left({\hat{x}}_{s}\left(t-1\right),u\left(t\right),{\hat{x}}_{p}^{-}\left(t\right)\right)$$

(32) $$P_{s}^{-}\left(t\right)\;=F_{s}\left(t\right)P_{s}\left(t-1\right)F_{s}^{T}\left(t\right)+Q_{s}$$

State update:

(33) $$K_{s}\left(t\right)\;=P_{s}^{-}\left(t\right)H_{s}^{T}\left(t\right){\left[H_{s}\left(t\right)P_{s}^{-}\left(t\right)H_{s}^{T}\left(t\right)+R_{s}\right]}^{-1}$$

(34) $${\hat{x}}_{s}\left(t\right)\;={\hat{x}}_{s}^{-}\left(t\right)+K_{s}\left(t\right)\left[y\left(t\right)-h\left({\hat{x}}_{s}^{-}\left(t\right)\right)\right]$$

(35) $$P_{s}\left(t\right)\;=P_{s}^{-}\left(t\right)-K_{s}\left(t\right)H_{s}\left(t\right)P_{s}^{-}\left(t\right)$$

Parameter update:

(36) $$K_{p}\left(t\right)\;=P_{p}^{-}\left(t\right)H_{p}^{T}\left(t\right){\left[H_{p}\left(t\right)P_{p}^{-}\left(t\right)H_{p}^{T}\left(t\right)+R_{p}\right]}^{-1}$$

(37) $${\hat{x}}_{p}\left(t\right)\;={\hat{x}}_{p}^{-}\left(t\right)+K_{p}\left(t\right)\left[y\left(t\right)-h\left({\hat{x}}_{s}^{-}\left(t\right)\right)\right]$$

(38) $$P_{p}\left(t\right)\;=P_{p}^{-}\left(t\right)-K_{p}\left(t\right)H_{p}\left(t\right)P_{p}^{-}\left(t\right)$$

where the parameter vector ${\hat{x}}_{p}={\left[C_{\alpha f}\;C_{\alpha r}\right]}^{T}$, state vector ${\hat{x}}_{s}={\left[v_{x}\;v_{y}\;v_{z}\;\dot{\phi }\;\dot{\theta }\;\dot{\psi }\;\phi \;\theta \right]}^{T}$; $P_{p}$ and $P_{s}$ are the error covariance matrices for parameters and states, respectively; $Q_{p}$ and $Q_{s}$ are the process noise covariance matrices for parameter and state estimators, respectively; $R_{p}$ and $R_{s}$ are the output noise covariance matrices for parameter and state estimators, respectively. $R_{p}$ and $R_{s}$ are the same because both the parameter and state estimator have same output y. $K_{p}$ and $K_{s}$ are the Kalman gain matrices for parameter and state estimators, respectively. $F_{s}$ and $H_{s}$ are the Jacobian matrices for the state and output equations, respectively, and are expressed as follows:

(39) $$F_{s}=\left[\begin{array}{ccc} \frac{\partial f_{1}}{\partial x_{1}} & \cdots & \frac{\partial f_{1}}{\partial x_{8}}\\ \vdots & \ddots & \vdots \\ \frac{\partial f_{8}}{\partial x_{1}} & \cdots & \frac{\partial f_{8}}{\partial x_{8}} \end{array}\right]$$

(40) $$H_{s}=\left[\begin{array}{ccc} \frac{\partial h_{1}}{\partial x_{1}} & \cdots & \frac{\partial h_{1}}{\partial x_{8}}\\ \vdots & \ddots & \vdots \\ \frac{\partial h_{5}}{\partial x_{1}} & \cdots & \frac{\partial h_{5}}{\partial x_{8}} \end{array}\right]$$

In Equations (36) and (38), $H_{p}$ is the Jacobian matrix of state output for the parameter estimator and can be expressed as:

(41) $$H_{p}=\frac{\partial y}{\partial {\hat{x}}_{p}}=\left[\begin{array}{c} \begin{array}{cc} \frac{\partial a_{x}}{\partial C_{\alpha f}} & \frac{\partial a_{x}}{\partial C_{\alpha r}} \end{array}\\ \vdots \\ \begin{array}{cc} \frac{\partial \theta }{\partial C_{\alpha f}} & \frac{\partial \theta }{\partial C_{\alpha r}} \end{array} \end{array}\right]$$

$R_{p}$ and $R_{s}$ can be determined by the engineer’s tuning based on sensor noise. $Q_{s}$ and $Q_{p}$ can also be determined by engineer’s tuning but $Q_{p}$’s square of each elements is tuned to approximately 1% of the initial values of the each actual parameter.

## 4. Results and Analysis

This section presents and discusses the experimental verification results of the algorithms mentioned in Section 3. Neural network and DEKF are discussed separately in Section 3.1 and Section 3.2, respectively. The performance is compared with that of commercial sensors.

### 4.1. Roll and Pitch Estimator (Neural Network)

#### 4.1.1. Dataset

Sensor data from real-car experiment data were used for training and validating the neural network. The neural network input data set contained information of a car’s chassis sensors, and the label data set of roll and pitch angles was obtained from the high-accuracy GPS-inertial navigation system (INS) RT-3002 (Oxford Technical Solutions Ltd., Bicester, UK). For training the neural network, a total of 176,259 data sets were used, which logged about 30 min at 10 ms intervals in various situations and offline validation was performed in scenarios as shown in Table 3 with the same vehicle. The software used Python and the framework used TensorFlow 1.6.

#### 4.1.2. Validation Result and Analysis

**Figure 5:**
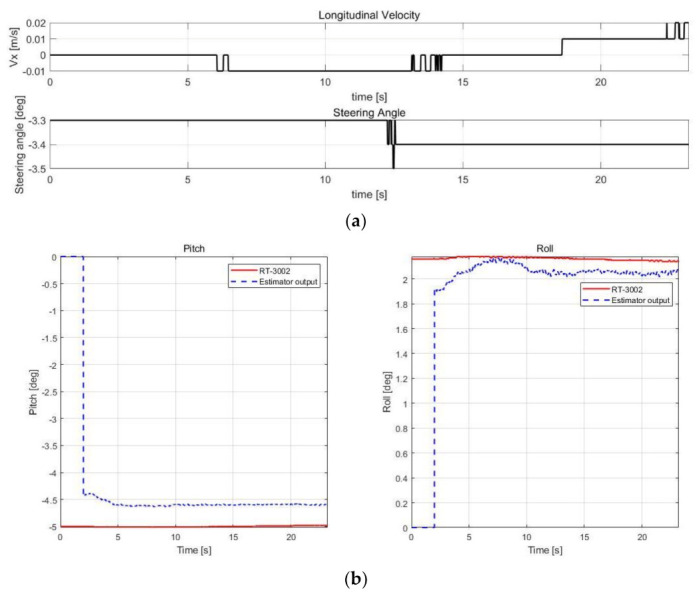
Validation results of Case 1. (**a**) Speed and steering profile. (**b**) Roll/pitch estimation results.

**Figure 6:**
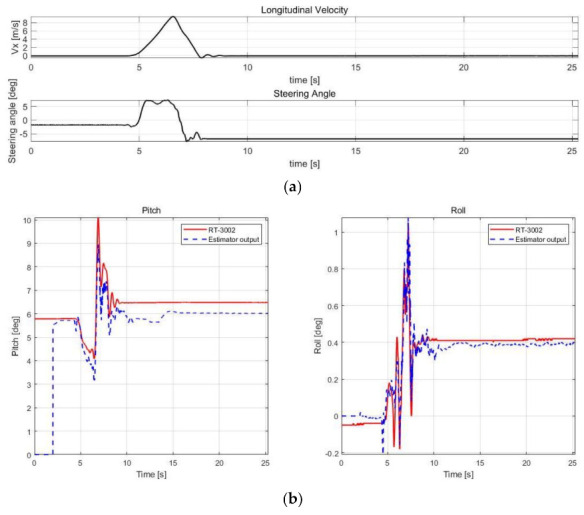
Validation results of Case 2. (**a**) Speed and steering profile. (**b**) Roll/pitch estimation results.

**Figure 7:**
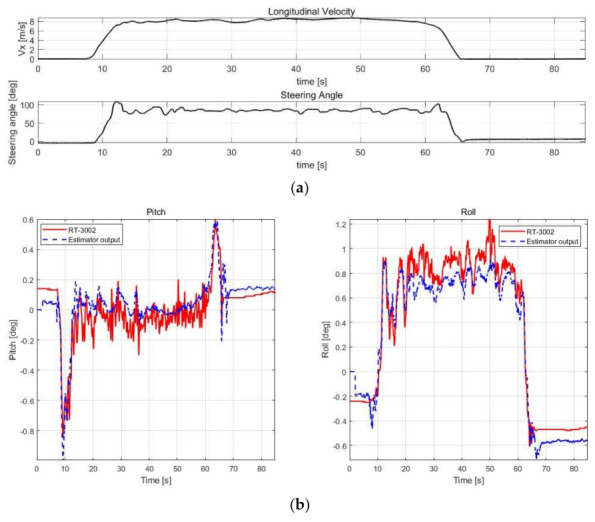
Validation results of Case 3. (**a**) Speed and steering profile. (**b**) Roll/pitch estimation results.

**Figure 8:**
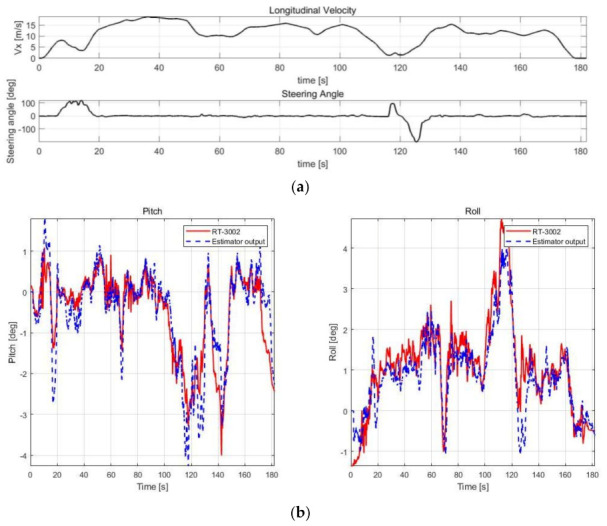
Validation results of Case 4. (**a**) Speed and steering profile. (**b**) Roll/pitch estimation results.

The estimation performance was validated using offline sensor data logged in various cases, as shown in Table 3. The root mean square error (RMSE) was calculated by comparison with RT-3002, which was treated as a reference, and with the datasheet of SST810, which is a commercial inclinometer sensor (Vigor Technology Co., Ltd., Xiamen, China). Figure 5, Figure 6, Figure 7 and Figure 8 show the scenario and roll/pitch estimation results for Case 1–4, respectively.

Table 4 shows the accuracy of the SST810 datasheet and RMSE calculation results of the estimation results for Case 1–4. Figure 5, Figure 6, Figure 7 and Figure 8 show that the value of pitch and roll angles between 0 and 2 s is fixed at zero. This is due to the structure described in Section 3.1.2, requiring 2 s of sequential data for the input; thus, the values in the initial 2 s cannot be calculated. The RMSE was therefore calculated in the time zone excluding the initial 2 s.

Case 1 shows that the roll RMSE is approximately 0.1° and the pitch RMSE is approximately 0.4°. There was some offset error, although the vehicle’s speed was zero, i.e., a stationary scenario. For commercial sensors, the error rate is 0.05° at the static scenario, but the estimation results show a larger error compared with the commercial sensors. The neural network estimator in this study uses data of only the chassis sensors of the vehicle; thus, the performance of sensors has a significant impact on the estimation performance. In particular, it can be expected that the IMU’s characteristic bias error affected the offset errors in the estimation results.

Cases 2–4 include scenarios in which rapid changes in vehicle attitude occur in areas where acceleration or deceleration occurs. The validation results show high estimation accuracy in these scenarios. In addition, the noise reduction effect compared to RT-3002 is noticeable in the 15–60 s duration for Case 3; thus, the neural network estimator can estimate stable output. The performance of the estimator is thus superior compared to the commercial sensors.

### 4.2. Acceleration and Angular Velocity Estimator (DEKF)

#### 4.2.1. Validation Environment

The acceleration and angle velocity estimator was validated by simulation using CarSim, which is a commercial vehicle simulator software. To create an environment similar to the actual vehicle, Gaussian white noise was added to the sensor value, as shown in Table 5. To ensure that the errors of the acceleration sensor due to roll and pitch have been corrected, simulations were conducted in the scenarios shown in Table 6.

#### 4.2.2. Validation Results and Analysis

**Figure 9:**
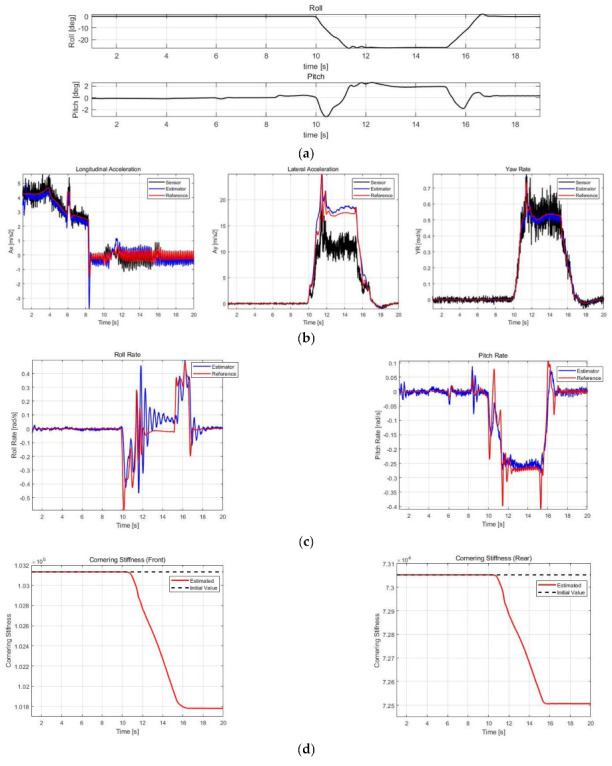
Simulation results of Case 1. (**a**) Roll/pitch profile. (**b**) $a_{x}$, $a_{y}$ and $\dot{\psi }$ estimation results. (**c**) $\dot{\phi }$ and $\dot{\theta }$ estimation results. (**d**) $C_{\alpha f}$ and $C_{\alpha r}$ estimation results.

**Figure 10:**
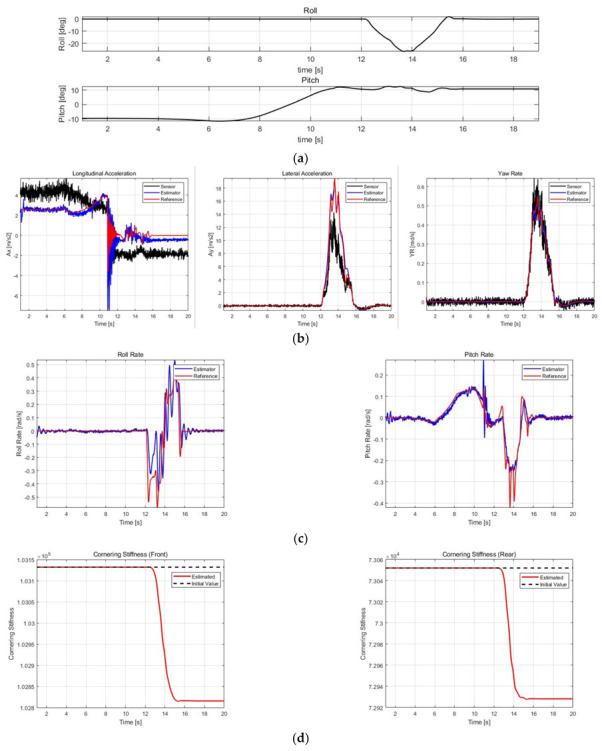
Simulation results of Case 2. (**a**) Roll/pitch profile. (**b**) $a_{x}$, $a_{y}$, and $\dot{\psi }$ estimation results. (**c**) $\dot{\phi }$ and $\dot{\theta }$ estimation results. (**d**) $C_{\alpha f}$ and $C_{\alpha r}$ estimation results.

**Figure 11:**
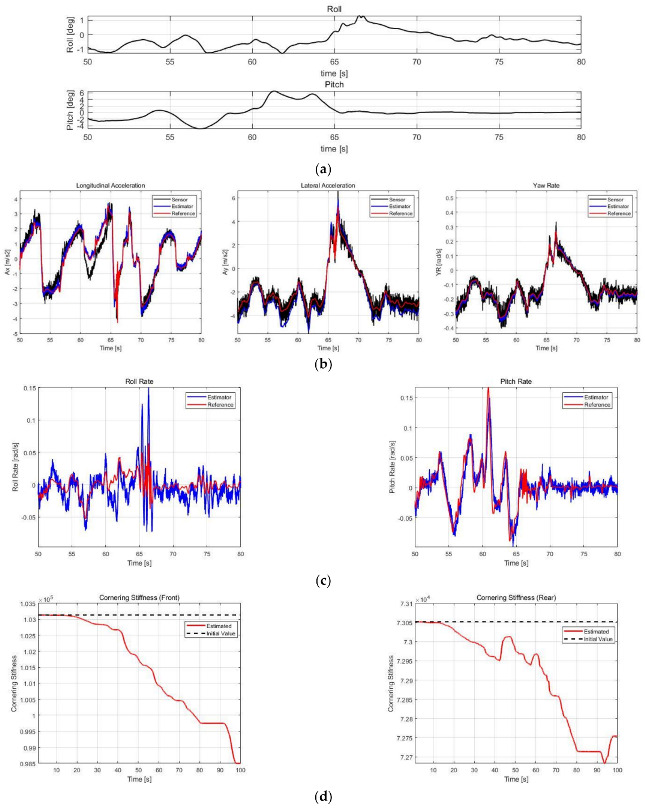
Simulation results of Case 3. (**a**) Roll/pitch profile. (**b**) $a_{x}$, $a_{y}$, and $\dot{\psi }$ estimation results. (**c**) $\dot{\phi }$ and $\dot{\theta }$ estimation results. (**d**) $C_{\alpha f}$ and $C_{\alpha r}$ estimation results.

**Figure 12:**
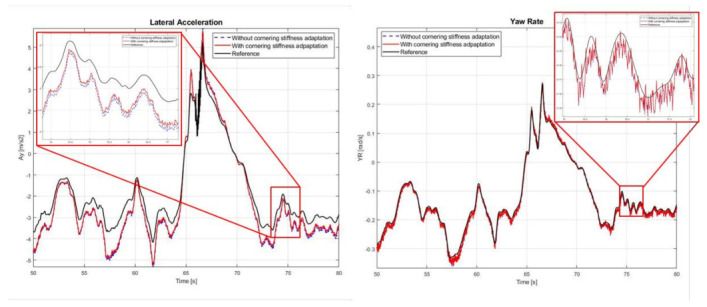
Effect of cornering stiffness estimation in Case 3.

The correctness performance of $a_{x}$, $a_{y}$, and $\dot{\psi }$ was validated by comparing IMU sensor values, DEKF estimates, and reference values, while the estimates of roll rates and pitch rates without sensors were validated by comparing with only the reference values. The RMSE of the estimated results is calculated based on the reference and compared with the datasheet of SMI860, which is a commercial six-dimensional IMU from BOSCH Co., Ltd. In addition, to validate the effects of DEKF’s parameter estimator, the estimated results of $C_{\alpha f}$ and $C_{\alpha r}$ for each case were noted, and the estimated results with and without cornering stiffness estimation for Case 3 were compared. Figure 9, Figure 10, Figure 11 and Figure 12 show the simulation scenarios and results of DEKF.

Table 7 shows the accuracy of the commercial sensor, obtained from its datasheet, and RMSE calculation results of the estimation values for case 1–3. In case 1, there was a fatal error of sensor value of $a_{y}$ due to roll angle, and case 2 exhibited considerably varying roll and pitch angles; therefore, the value of $a_{x}$ and $a_{y}$ from sensors may not be accurate. Figure 9b and Figure 10b confirm that these errors in sensor values are successfully corrected to obtain estimates close to the reference. In addition, in normal driving scenarios, as in the 60–65 s duration in Case 3, an $a_{x}$ sensor error by pitch is noted, which has also been successfully corrected. Furthermore, in all cases, filter effects that reduce noise from existing sensors can be checked through $a_{x}$, $a_{y}$, and $\dot{\psi }$. Compared with a commercial sensor, $a_{x}$ and $a_{y}$ show similar accuracy, whereas $\dot{\psi }$ shows a significantly higher accuracy. However, in the case of commercial sensors, there may be errors caused by roll and pitch angles; therefore, DEKF can have commercial sensor level or higher accuracy even though correcting those errors.

The roll and pitch rates could be estimated because they were included in the state vector, although the sensor values were not included. The roll rate accuracy was occasionally lower than that of commercial sensors depending on the case, and in the case of pitch rate, it is not comparable because there is no output of commercial sensors, but it can be confirmed from the RMSE values that the estimated values have a fairly high accuracy.

Figure 9d, Figure 10d and Figure 11d show the estimated cornering stiffness. Figure 12 and Table 8 show the results with and without cornering stiffness estimation in Case 3, which can improve accuracy by approximately 3–5%, particularly with a greater effect on $a_{y}$.

## 5. Conclusions and Future Work

In this paper, we proposed a CNN-based neural network to estimate the roll and pitch angles of a vehicle. A DEKF was used to correct the gravitational effect caused by the roll and pitch for estimating the exact acceleration and angular velocity.

By using the vehicle’s chassis sensor data as a time series, the neural network could estimate the roll and pitch angles of the vehicle without a GPS or six-dimensional IMU. Based on the estimated roll and pitch angels, we designed an extended Kalman filter (EKF) using the 6-DOF vehicle model. Another EKF was designed, and the two EKFs were used to estimate the cornering stiffness. We then constructed the DEKF.

Using experimental data obtained using a real car, the proposed roll and pitch estimator was validated, and the DEKF was validated in the CarSim simulation environment. The roll and pitch estimator showed an improved performance compared to the commercial sensors in dynamic scenarios and also reduced the noise. However, the performance in static scenarios was weaker. The acceleration and angular velocity estimator could effectively correct the acceleration sensor error due to roll and pitch with a de-noising effect. In addition, the roll and pitch rates that could not be obtained from sensors could be estimated with significant accuracy. By comparing the results before and after including the cornering stiffness, we found that the accuracy is improved if the cornering stiffness is considered.

On the other hand, our work has limitations and challenges that should be further discussed. We plan to consider fusion with other algorithms to improve the attitude estimation performance in static scenarios. In addition, the proposed method has not been checked in case of a change in the vehicle weight. It will also be necessary to verify the performance of the algorithm due to changes in vehicle weight. Furthermore, our proposed algorithm is hard to apply as an embedded system in vehicle because the neural network has large capacity. The future work should be conducted to enable algorithm to operate as real-time in vehicle through simplification and optimization.

## Figures and Tables

**Table 1:** Literature review summarizing.

References	Methodology	Model
[8]	Linear observer	IMU kinematic model
[9]	Kalman filter	IMU + Vehicle dynamics (bicycle model and wheel model)
[10,11,12,13]	Kalman filter	IMU external acceleration model
[14,15,16]	Kalman filter	IMU + GPS model
[17]	Sliding mode observer	IMU + GPS model
[18]	Dynamic observer	Vehicle roll dynamics
[19]	Kalman filter	Vehicle roll dynamics
[20]	Dual Kalman filter	Vehicle roll dynamics
[21,22]	Linear observer	Vehicle lateral dynamics (bicycle model)
[23,24]	Linear observer, Sliding mode observer	Vehicle lateral dynamics (bicycle model) + IMU
[25,26,27]	Kalman filter + neural network	Vehicle roll dynamics + fully connected layer
[28]	Neural network	Fully connected layer

**Table 2:** Selected features considering sensor usability.

Feature Name	Description
Ax	Vehicle longitudinal acceleration from IMU
Ay	Vehicle lateral acceleration from IMU
Yaw Rate	Vehicle yaw rate from IMU
Brake Pres	Brake pressure from the master cylinder
Str Angle	Steering wheel angle
Throttle	Engine throttle valve opening degree (0~1)
ΣWheelSpd (Vx)	Sum of the wheel rotation speed

**Table 3:** Roll/pitch estimator validation scenario.

	Condition	Description
Case 1	-	A stationary situation on the uphill
Case 2	Acceleration ±0.5 g or higher Steering ±5 deg or higher	Rapid acceleration with steering on downhill slope-quick deceleration
Case 3	Steering ±50 deg or higher Yaw rate ±30 deg/s or higher	Accelerate-turn-deceleration with steering on flat road
Case 4	-	Common driving

**Table 4:** Root mean square error (RMSE) calculation results of roll/pitch estimator and accuracy of SST810 datasheet.

	Case 1		Case 2		Case 3		Case 4	
	Roll	Pitch	Roll	Pitch	Roll	Pitch	Roll	Pitch
RMSE (deg)	0.1133	0.4188	0.0573	0.5422	0.1359	0.0958	0.5140	0.5283
Commercial sensor accuracy (deg) ^1^	≤±0.05 (static situation)		≤±0.5 (dynamic situation)					

^1^ From SST810 inclinometer datasheet of Vigor Technology Co., Ltd.

**Table 5:** Sensor configuration for simulation.

Sensor	Noise	Unit
IMU ($a_{x},\;a_{y}$)	0.1 (RMS) + 10 (%)	m/s^2^
IMU ($\dot{\psi }$)	0.01 (RMS) + 10 (%)	rad/s
Steering angle	0.05 (RMS) + 10 (%)	rad
Engine torque	7 (RMS)	N·m
Brake pressure	0.05 (RMS)	MPa

**Table 6:** Acceleration/angular velocity estimator validation scenario.

	Condition	Description
Case 1	Roll ±30 deg or lower Pitch ±3 deg or lower	U-turn with 30 degrees of bank angle
Case 2	Roll ±30 deg or lower Pitch ±10 deg or lower	Sharp turn at 30 degrees of bank angle after 10 degrees of uphill and downhill
Case 3	-	Common driving

**Table 7:** RMSE calculation results of DEKF and accuracy obtained from SMI860 datasheet.

		Accuracy	
		DEKF (RMSE)	Commercial Sensor ^1^
$a_{x}$ (m/s^2^)	Case 1	0.4325	≤±0.5
	Case 2	0.8075	
	Case 3	0.3087	
$a_{y}$ (m/s^2^)	Case 1	0.8232	≤±0.5
	Case 2	0.4204	
	Case 3	0.5085	
$\dot{\psi }$ (deg/s)	Case 1	0.7391	≤±3
	Case 2	0.3953	
	Case 3	0.5844	
$\dot{\phi }$ (deg/s)	Case 1	5.8499	≤±2
	Case 2	5.1394	
	Case 3	1.0542	
$\dot{\theta }$ (deg/s)	Case 1	1.9251	- ^2^
	Case 2	1.7475	
	Case 3	0.6704	

^1^ From SMI860 IMU datasheet of BOSCH Co., Ltd. ^2^ SMI860 cannot sense the pitch rate.

**Table 8:** RMSE of with and without cornering stiffness estimation in Case 3.

	RMSE	
	Without Cornering Stiffness Estimation	With Cornering Stiffness Estimation
$a_{y}$ (m/s^2^)	0.5541	0.5085
$\dot{\psi }$ (deg/s)	0.6131	0.5844

**Figure A1:**
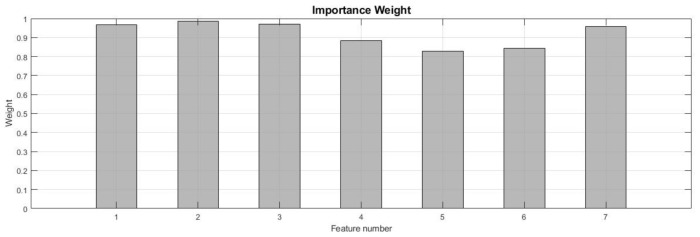
Result of feature importance weight analysis.

## Data Availability

Restrictions apply to the availability of these data. Data was obtained from Hyundai Motor Company and are available with the permission of Autonomous Driving Center of Hyundai Motor Company R&D Division.

## Appendix A. Feature Importance Analysis

**Table A1:** Feature name of each number.

Feature Number	Feature Name
1	Ax
2	Ay
3	Yaw Rate
4	Brake Pressure
5	Steering Angle
6	Engine Throttle
7	ΣWheel Speed (Vx)

All features have an importance weight higher than 0.8; therefore, feature reduction is not performed.

## Appendix B. Hyperparameter of Neural Network

**Table A2:** Details of neural network.

Part	Layer	Name	Filter Size	Activation	Output Size
CNN part	1-layer	Fully connected		Leaky ReLU	(200 × 200 × 1)
	2-layer	Convolution layer	(3 × 3 × 1) 8EA ^1^	Leaky ReLU	(200 × 200 × 8)
	3-layer	Convolution layer	(3 × 3 × 8) 16EA ^1^	Leaky ReLU	(200 × 200 × 16)
	4-layer	Convolution layer	(3 × 3 × 16) 32EA ^1^	Leaky ReLU	(200 × 200 × 32)
	5-layer	Convolution layer	(5 × 5 × 32) 32EA ^2^	Leaky ReLU	(40 × 40 × 32)
	6-layer	Fully connected		Leaky ReLU	(256 × 1)
FCL part	1-layer	Fully connected		Leaky ReLU	(256 × 1)
	2-layer	Fully connected		Leaky ReLU	(1024 × 1)
	3-layer	Fully connected		Leaky ReLU	(1024 × 1)
	4-layer	Fully connected		Leaky ReLU	(256 × 1)
Final Layer	Final layer	Fully connected			(2 × 1)

^1^ 1 × 1 stride was used. ^2^ 5 × 5 strides were used.
